# Helminths of white stork *Ciconia ciconia* in north-eastern Poland

**DOI:** 10.1038/s41598-025-32649-9

**Published:** 2026-01-12

**Authors:** Agnieszka Tylkowska, Agata Stapf, Izabella Rząd, Rusłan Sałamatin, Remigiusz Panicz, Witold Jeżewski, Adam Kaczmarek, Piotr Eljasik

**Affiliations:** 1 Department of Biology of Animal Environment, Institute of Animal Science, Warsaw University of Life Sciences, Ciszewskiego 8, Warsaw, 02-786 Poland; 2 Department of Biological Sciences, Faculty of Sport Sciences in Gorzów Wielkopolski, Estkowskiego 13, University of Physical Education, Poznań, Poland; 3 Institute of Marine and Environmental Sciences, University of Szczecin, Wąska 13, Szczecin, 71-415 Poland; 4 Molecular Biology and Biotechnology Centre, University of Szczecin, Wąska 13, Szczecin, 71-415 Poland; 5 Department of Microbiology and Parasitology, Faculty of Medicine. Collegium Medicum, Cardinal Stefan Wyszynski University in Warsaw, Wóycickiego 1/3, Warsaw, 01-938 Poland; 6 Department of General Biology and Parasitology, Medical University of Warsaw, Chałubińskiego 5, Warsaw, 02-004 Poland; 7 Faculty of Food Science and Fisheries, West Pomeranian University of Technology in Szczecin, Kazimierza Królewicza 4, Szczecin, 71-550 Poland; 8 Witold Stefański Institute of Parasitology, Polish Academy of Sciences, Twarda 51/55, Warsaw, 00-818 Poland; 9 Department of Parasitology and Vector-Borne Diseases, National Institute of Public Health NIH – National Research Institute, Chocimska 24, Warsaw, 00- 791 Poland

**Keywords:** Ciconia ciconia, White stork, Parasites, Helminths, Poland, Ecology, Ecology, Zoology

## Abstract

The aim of the study was to determine the structure of helminth communities of White Stork in north-eastern Poland in the years 2015–2018. The study material comprised 61 White Storks *Ciconia ciconia* from within the borders of Biebrza National Park. Seven parasite taxa were detected in the storks. The cestode *Dictymetra riccii*, the nematode *Dispharynx* cf. *nasuta*, and acanthocephalans are new parasites for storks in Poland. Prevalence of helminths was 32.8%, with a mean intensity of 12.2, in a range from 1 to 60 and with mean abundance of 3.8. The biodiversity of the component community expressed by Simpson’s diversity index was 0.639, and dominance expressed by the Berger–Parker index was 0.485. The dominant species in the component community was the cestode *Dictymetra discoidea*. The average biodiversity of infracommunities, expressed by Brillouin’s diversity index, was 0.07 ± 0.13. The greatest species richness of helminths in storks was recorded in July (five species). The greatest intensity of infection was noted in the case of infection with *Tylodelphys excavata* in June. The prevalence of the cestode *Dictymetra discoidea* in adult storks (35.5%) was significantly higher than in juvenile storks (4.17%) (chi^2^ = 7.78, *p* = 0.005).

## Introduction

The White Stork *Ciconia ciconia* (Linnaeus, 1758) belongs to the order Ciconiiformes and the family Ciconiidae. It inhabits wet lowlands with clumps of trees and medium-high vegetation on the Iberian Peninsula and in southern Africa, Asia Minor, Central Asia, and Central and Eastern Europe^1^. The breeding territory of the White Stork encompasses most of Europe, Asia Minor, and North Africa. When storks which nest in Poland migrate to Africa, they choose an eastern route, via Turkey and the Bosporus, whereas birds from Western Europe fly over Spain and Gibraltar. The White Stork is a species protected under the Nature Conservation Act and the Bonn, Bern, and Ramsar Conventions, and it is also named in the European Union Birds Directive. It inhabits agricultural landscapes and is mainly associated with wetlands. It nests in colonies, usually in open meadows, especially wet or periodically flooded grasslands. The species is synanthropic and often nests near human settlements. Storks have been observed to forage on rubbish dumps near cities^2^. Colonies can also be found away from human settlements, in wooded areas in river floodplains. White Storks feed on insects, fish, amphibians, reptiles, small mammals, and small birds; the basis of their diet is arthropods and earthworms^3^. They find most of their food on the ground, amid low vegetation, and in shallow water. Their diet varies depending on the time of year, region, and food availability. Animals making up the diet of White Storks, such as rodents, amphibians, and fish, can be a source of pathogens^4,5^.

Knowledge of parasites of White Storks is insufficient, and the structure of their helminth communities is as yet unknown. The best known parasites are those that cause pathological symptoms and affect the health and condition of the birds. Intestinal parasites occurring with high intensity of infection can cause intestinal obstruction^6^. Trematodes *Chaunocephalus ferox* (Rudolphi, 1795) (Echinostomatidae) cause changes in the intestines which pose a threat to the health of storks and often have lethal consequences^7,8^. Parasites can directly affect the birds’ ability to fly, especially in juveniles and in older birds with chronic parasitic infections^9,10^. In addition to helminths, *Toxoplasma gondii* Nicolle & Manceaux, 1908 antibodies have also been detected in White Storks^11^. *T. gondii* is a parasite of zoonotic importance; one third of the world’s human population is believed to be infected with this protozoan.

Recent years have seen a decline in the number of storks at breeding sites. This decline can be ascribed to habitat changes, excessive use of pesticides, electrocution, and road accidents^12^. According to Höfle et al.^13^ and Santoro et al.^8^, the health condition and welfare of storks depends mainly on environmental conditions. In Poland, the most stork colonies are found in the northeast and east of the country. Numerous colonies are distributed in villages and towns around the Biebrza Marshes, by the Narew River, and in the Bug and San River valleys. Monitoring of Flagship Bird Species (MFGP) by the Chief Inspectorate for Environmental Protection shows that the stork population in Poland has decreased by 0.4% a year for the last 14 years. The situation is confirmed by data from the report of the Prioritised Action Framework (PAF) for the Natura 2000 network for the 2014–2020 UE financing period, according to which the trend is downward. The White Stork is considered an umbrella species, i.e. one whose protection benefits many other co-occurring species and their habitats. The White Stork is a species which responds directly to changing availability of food resources, usually due to environmental changes. The main threats facing these birds are a decrease in the area of their feeding grounds and a decrease in the abundance of prey, due to river regulation, management of river valleys other than as grassland, land amelioration, and the intensification of agriculture. Changes in the natural environment resulting from direct human activity as well as the warming climate affect the relationships between the White Stork and its parasites, as observed in many other parasite–host systems. Due to changes in their habitats, especially during migration, birds search for food in new places and thus can acquire different species of helminths^14^.

The aim of the study was to determine the community structure of helminths of White Stork in north-eastern Poland, from their arrival to the breeding grounds in spring until their departure in late summer. The study was undertaken in view of the lack of up-to-date knowledge of parasites of storks in Poland and Europe, which could help to assess the condition of the population. The White Stork is identified with Poland around the world, functions as its ‘national bird’, and is a direct link between nature and human beings.

## Methods

The study material comprised 61 White Storks *Ciconia ciconia* from within the borders of Biebrza National Park (BPN) in north-eastern Poland. North-eastern Poland is considered to be the coldest part of the country (apart from the mountains). The climate is near-continental, with sub-boreal elements. It is characterized by a long winter, a short transition from winter to spring, and the shortest growing season in the country (outside the mountains). The average annual temperature is one of the lowest in lowland regions in Poland. Biebrza National Park contains communities of aquatic, wetland, peatland, and shoreline vegetation, as well as forest communities.

The storks used for parasitological analysis were obtained from the wildlife rehabilitation centre in BPN in the years 2015–2018. Parasite samples were taken from dead birds. No birds were intentionally killed for parasitological analysis. The birds had not been previously dewormed (Table 1).

**Table 1:** Study material – numbers of storks examined for parasites from April to August in the years 2015–2018, obtained from Biebrza National park (*N* = 61 storks examined).

	Adults		Juveniles		Chicks		Total
Month	F	M	F	M	F	M	
April	2	3	0	0	0	0	**5**
May	3	3	0	0	0	0	**6**
June	1	4	1	0	2	1	**9**
July	0	8	11	5	1	2	**27**
August	3	4	7	0	0	0	**14**
Total	**9**	**22**	**19**	**5**	**3**	**3**	**61**

*F* Female, *M* Male.

The oral cavity and coelom as well as the respiratory, digestive, reproductive, and urinary systems of the storks were examined for parasites. The parasites found were preserved in 75% ethyl alcohol. Permanent slides of trematodes were prepared, which were stained with alum carmine, cleared in clove oil, and mounted in Canada balsam. Scolices of cestodes were cleared in Faure’s medium. Strobili were stained with acetocarmine, and permanent slides were prepared from them. They were cleared in creosote and mounted in Canada balsam. Nematodes and acanthocephalans were cleared in glycerine. The parasites were observed, their morphology was described, and morphometric measurements were made using biological microscopes, at magnification from 10x to 400x. For taxonomic identification of helminths on the basis of morphological characters and morphometric measurements, keys and original works were used^4,15–21^. Body fragments of acanthocephalans were used for genetic analysis to confirm their genus or species.

### Molecular identification of parasite specimens

For molecular identification of the two acanthocephalan specimens, they were air-dried under ambient conditions for 10 min. The High Pure PCR Template Preparation Kit (Roche, Switzerland) was used for DNA extraction according to the manufacturer’s instructions. The quantity and quality of the extract were determined by spectrophotometric measurements using the NanoDrop 2000 spectrophotometer (Thermo Scientific, USA) and electrophoresis in a 1.5% agarose gel. Due to the low DNA concentration (< 5 ng/µl) and poor sample quality (signs of degradation were apparent on the agarose gel), the use of several primer pairs in different configurations and extensive optimization (e.g. adjusted PCR conditions, reamplification, nested PCR, and semi-nested PCR) failed to amplify the COX1, 18 S rRNA and 28 S rRNA regions (Table 2) on both specimens. Amplification was partially successful only with the SSU-rDNA-F and SSU-rDNA-R primers for 18 S rRNA under conditions recommended by García-Varela et al.^22^. Sanger sequencing revealed only partial 18 S rRNA sequences (approximately 400 bp of an expected 1800 bp) due to background noise (multiple reads); these sequences were not used for specimen identification. Based on the raw reads, a new primer set was designed using Primer 3^23^. Amplification was conducted on the Mastercycler (Eppendorf) under the following conditions: one step of 5 min at 94 °C followed by 45 cycles at 94 °C for 30 s, 55 °C for 30 s, 72 °C for 30 s, and final extension at 72 °C for 7 min. Reactions were prepared with the GoTaq PCR kit (Promega), which includes 5 µl of Green GoTaq Flexi Buffer, 2.5 µl of MgCl_2_ (25 mM Solution), 0.5 µl of PCR Nucleotide Mix (10 mM), 0.125 µl of GoTaq DNA Polymerase (5 u/l), 0.5 µM of each primer, and 3 µl of DNA template in a final volume of 25 µl. The results of end-point PCR reactions were assessed by 1.5% gel electrophoresis, and positive samples were sequenced on both strands using direct Sanger sequencing by Genomed (Warsaw) to eliminate false-positive results. Raw sequences were processed, aligned, and checked using Geneious v.11.1.5 sequence editing software (created by Biomatters, available from http://www.geneious.com). Finally, all sequences were searched for matches against the GeneBank database using BLASTn.

**Table 2:** Methods of genetic analysis of fragments of acanthocephalans from the intestines of white Stork.

Primer name	Primer sequence 5’->3’	Target region	References
L18S1	TTTATGGATCCGCGGCTTAG	3’ end of the 18 S gene/5’ end of the 28 S gene	^24^
L28SR1	AACCAAATGGTCACAGGCTT		
NLF1	GACTCCTTTACTGGTTTGATCG	cytochrome oxidase subunit I	^25^
NLF2	GCACATAATGAAAATGAGCC		
SSU-rDNA-F	AGATTAAGCCATGCATGCGT	18 S rRNA	^22^
SSU-rDNA-R	AACTTTTCGTTCTTGATTAATG		
LSU rDNA-F	GAGTTCACAAGTGCGTGAAAC	28 S rRNA	^26^
LSU rDNA-R	CTTCGCAATGATAGGAAGAGCC		
Kok05-412 F	GGCGCGCAAATTACCCAATT	18 S rRNA	This study
Kok05-736R	GCTCTTCACCGAGACACACA		

### Parasitological indicators

The following parasitological indicators were determined: prevalence (P), as the percentage of infected storks among all those examined; mean intensity (MI), as the average number of parasites per infected stork; range of intensity (Min–Max), i.e. the minimum and maximum number of parasites in individual storks, and mean abundance (MA), as the average number of parasites per examined stork, including both infected and uninfected storks^27^. Quantitative parameters were estimated using the values of the confidence limits. The computations were performed in Quantitative Parasitology 3.0 software (QP3.0; http://www.zoologia.hu/qp/). Prevalence and intensity of infection were compared between the study months and between the following groups:

- age groups of storks (adults, juveniles, and chicks).
- all females (31) and all males (30).

Storks three years and older were considered to be adults. Juveniles were defined as feathered birds which have left the nest, born in the year of the study. Chicks were downy, had not left the nest, and were born in the year of the study.

Prevalence was compared using the chi square (chi^2^) test. Intensity of infection and relative density were compared using the non-parametric Mann–Whitney *U* test, as compliance with the normal distribution could not be confirmed due to the small sample sizes in the groups of storks. The computations were carried out in Statistica 12.

The structure of the helminth community was defined using indices according to Magurran^28^, as follows: (1) determination of the biodiversity of the component community using Simpson’s diversity index (1-D); 2) determination of dominance using the Berger–Parker dominance index, with specification of the dominant species in the community; and (3) determination of the biodiversity of infracommunities of helminths using Brillouin’s diversity index. Community indices were calculated only for birds infected with helminths. The component community of parasites is the set of all infracommunities in a given host population – in this case, the set of infracommunities of helminths in the sample of 61 storks. An infracommunity is the set of infrapopulations of all parasite species in the body of a single host – in this case, the group of helminth species found in the body of a single stork. An infrapopulation is a component of the population of a single parasite species at the level of a single host individual – in this case, the community of individuals of a single parasite species in a single stork. The dominant species is the species represented by the most individuals in the component community. These indices were calculated using Past v.2.11^29^.

## Results

Seven parasite taxa were recorded in the storks, including three species of trematodes (Digenea), two species of cestodes (Cestoda), one species of nematodes (Nematoda), and one genus of acanthocephalans (Acanthocephala). The cestode *Dictymetra riccii* (Fuhrmann & Baer, 1943) Clark, 1952, the nematode *Dispharynx* cf. *nasuta* (Rudolphi, 1819), and acanthocephalans are new parasites for storks in Poland.

### Parasitological and ecological indicators

The prevalence of helminths was 32.8%, with a mean intensity of 12.2 parasites, in a range from 1 to 60 and with mean abundance of 3.8. The prevalence of cestodes was the highest among the helminths, while that of nematodes was the lowest. The mean intensity of trematodes was highest among the helminths, while the mean abundance of trematodes and cestodes was similar and higher than that of nematodes and acanthocephalans. The most storks were infected by the cestode *Dictymetra discoidea* (van Beneden, 1868) Spasskaya & Shumilo, 1971, but the number of parasites in storks was highest in the case of the trematode *Tylodelphys excavata* (Rudolphi, 1803) (Table 3).

**Table 3:** Basic parasitological indicators in 61 storks from Biebrza National Park, examined in 2015–2018.

Helminths	*n*	*P* [%] (95% CL)	MI [mean] (95% CL)	MI [Min-Max]	MA [mean] (95% CL)	Location of parasites
*Chaunocephalus ferox* (Rudolphi, 1795)	6	9.8 (3.7–20.2)	6.2 (2.3–15.2)	1–21	0.6 (0.16–1.95)	intestine
*Tylodelphys excavata* (Rudolphi, 1803)	2	3.3 (0.4–11.3)	36.5 (13.0–36.5)	13–60	1.2 (0.00–4.4)	intestine
*Cathaemasia hians* (Rudolphi, 1809)	1	1.6 (0.0–8.8)	5.00 (0.0–0.0)	-	0.1 (0.0–0.3)	oesophagus
**Total Digenea**	**9**	**14.7** **(7.0–26.2)**	**12.8** **(5.0–31.6)**	**1–60**	**1.9** **(0.5–5.9)**	
*Dictymetra discoidea* (van Beneden, 1868) Spasskaya & Shumilo, 1971	12	19.7 (9.4–29.0)	10.2 (6.3–16.2)	1–30	1.8 (0.8–3.7)	intestine
*Dictymetra riccii* (Fuhrmann & Baer, 1943) Clark, 1952	1	1.6 (0.0–8.8)	1.0 (0.0–0.0)	-	0.0 (0.0–0.0)	intestine
**Total Cestoda**	**12**	**19.7** **(9.4–30.0)**	**10.3** **(6.5–16.7)**	**1–30**	**1.9** **(0.8–3.7)**	
*Dispharynx* cf. *nasuta* (Rudolphi, 1819)	1	1.6 (0.0–8.8)	1.0 (0.0–0.0)	-	0.0 (0.0–0.1)	oesophagus
**Total Nematoda**	**1**	**1.6** **(0.0–8.8)**	**1.0** **(0.0–0.0)**	**-**	**0.0** **(0.0–0.1)**	
*Centrorhynchus* spp.	2	3.3 (0.4–11.3)	1.0 (0.0–0.0)	1–1	0.0 (0.0–0.1)	intestine
**Total Acanthocephala**	**2**	**3.3** **(0.4–11.3)**	**1.0** **(0.0–0.0)**	**1–1**	**0.0** **(0.0–0.1)**	
**Total helminths**	**20**	**32.8** **(21.3–46.0)**	**11.6** **(7.5–20.6)**	**1–60**	**3.8** **(2.1–7.3)**	

*n* Number of infected birds, *P* Prevalence, *MI* Mean intensity of infection, *Min-Max* Range of intensity of infection, *MA* Mean abundance of helminths, *95% CL* 95% confidence limits.

A total of 231 helminth specimens were found in infected storks. The biodiversity of the component community expressed as Simpson’s diversity index was 0.639, and dominance expressed as the Berger–Parker index was 0.485. The dominant species in the component community was the cestode *Dictymetra discoidea*. The average biodiversity of the infracommunities, expressed as Brillouin’s index, was 0.1 ± 0.1. The average number of helminth species per infracommunity was 1.3 ± 0.5, in a range from 1 to 2 species, and the average number of parasite specimens per infracommunity was 12.2 ± 13.9, in a range from 1 to 60.

### Comparison of parasitological indicators between months of the study

The number of parasite species was lower from April to June than in July and decreased again in August. There were no significant differences in prevalence between months of the study, but the intensity of infection with helminths markedly decreased from April to July. The highest prevalence of helminths was noted in May and was caused by the presence of the cestode *D. discoidea*. The highest intensity of infection was noted for infection with *T. excavata* in June (Table 4).

**Table 4:** Comparison of parasitological indicators in the months of the study, from April to August (the number of storks examined is given in brackets).

Helminths	April (5)		May (6)		June (9)		July (27)		August (14)	
	*P.n* (%)	MI (Min – Max)	*P.n* (%)	MI (Min – Max)	*P.n* (%)	MI (Min – Max)	*P.n* (%)	MI (Min – Max)	*P.n* (%)	MI (Min – Max)
*Chaunocephalus ferox*	1 (20.0)	1	‒	‒	1 (11.1)	6	1 (3.7)	5	3 (21.4)	8.3 1–21
*Tylodelphys excavata*	‒	‒	‒	‒	1 (11.1)	60	1 (3.7)	13	‒	‒
*Cathaemasia hians*	1 (20.0)	5	‒	‒	‒	‒	‒	‒	0 (0.0)	‒
*Dictymetra discoidea*	1 (20.0)	30	4 (66.7)	9.7 (6–15)	1 (11.1)	20	4 (14.8)	2.7 1–5	2 (14.3)	7.5 (5–10)
*Dictymetra riccii*	‒	‒	1 (16.7)	1	‒	‒	‒	‒	‒	‒
*Dispharynx* cf. *nasuta*	‒	‒	‒	‒	‒	‒	1 (3.7)	1	‒	‒
*Centrorhynchus* spp.	‒	‒	‒	‒	‒	‒	1 (3.7)	1	1 (7.1)	1
Total helminths	2 (40.0)	18.0 (5–31)	4 (66.7)	10.0 (7–15)	3 (33.3)	43.0 (6–60)	6 (22.2)	5.6 (2–13)	5 (35.7)	8.2 (1–22)
Number of helminth species	3		2		3		5		3	

### Comparison of parasitological indicators in age groups of storks, by sex

Comparison of parasitological indicators in age groups broken down by sex is presented in Table 5. Prevalence of helminths was highest in female chicks, and the intensity of infection with helminths was also highest in a female chick (Table 5). Differences in the prevalence and intensity of infection with helminths (total) between adults, juveniles and chicks were not statistically significant.

**Table 5:** Prevalence (%) and intensity of infection with helminths in white stork by age and sex.

Age of storks	Adults		Juveniles		Chicks	
Sex of storks	**M**	**F**	**M**	**F**	**M**	**F**
N/n	8/22	4/9	2/5	4/19	0/3	2/3
Digenea						
P [%]	9.1	‒	20.0	21.0	‒	66.7
MI (Min-Max)	3.0 (1–5)	‒	13.0 (13)	7.7 (1–22)	‒	33.0 (6–60)
Cestoda						
P [%]	31.8	44.4	20.0	‒	‒	‒
MI (Min-Max)	8.8 (1–30)	13.3 (8–20)	2 (2)	‒	‒	‒
Nematoda						
P [%]	‒	‒	20.0	‒	‒	‒
MI (Min-Max)	‒	‒	1.0	‒	‒	‒
Acantocephala						
P [%]	4.5	‒	‒	5.3	‒	‒
MI (Min-Max)	1.0	‒	‒	1.0	‒	‒
Total						
P [%]	36.4	44.4	40.0	26.3	‒	66.7
MI (Min-Max)	8.6 (2–31)	13.5 (9–20)	8.5 (3–14)	6.6 (1–22)	‒	33.6 (6–60)

*N* Number of storks examined, *n* Number of infected birds.

Among the age groups of storks (adults, juveniles, and chicks), the prevalence of the cestode *Dictymetra discoidea* was higher in adult storks (*n* = 11, prevalence 35.5%) than in juveniles (*n* = 1, prevalence = 4.2%) and chicks (prevalence = 0.0%), and the difference in prevalence between adults and juveniles was statistically significant (chi^2^ = 7.78, *p* = 0.005). Intensity of infection, for which the differences between adults, juveniles, and chicks were tested by the Mann–Whitney *U* test, did not differ statistically significantly between stork age groups. For helminth species other than *D. discoidea*, statistical tests were not performed because their prevalence in individual age groups was too low. Comparison of parasitological indicators in all female and male storks, irrespective of age, showed no statistically significant differences between females and males in overall prevalence (of all helminths combined), the prevalence of individual helminth species, or the mean intensity of infection.

### Morphological and morphometric description of a new nematode species for storks, ***Dispharynx*** cf. ***nasuta*** (Rudolphi, 1819) Stiles & Hassall, 1920 (Acuariidae)

**Fig. 1:**
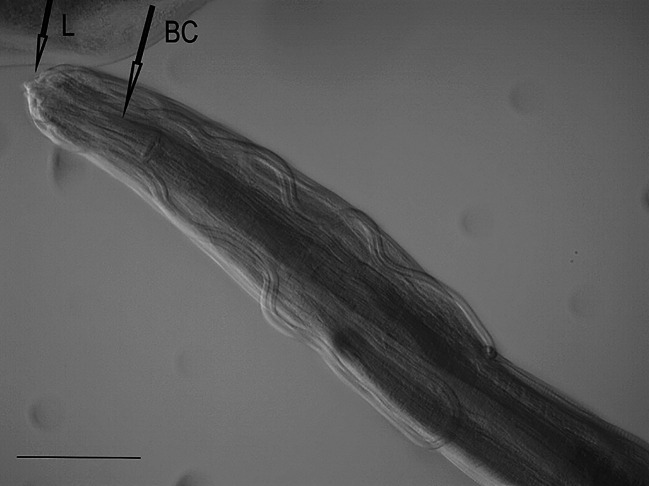
*Dispharynx* cf. *nasuta* from White Stork *Ciconia ciconia* (Poland), anterior end of coelom in female specimen. BC – buccal capsule, L – labia.

**Fig. 2:**
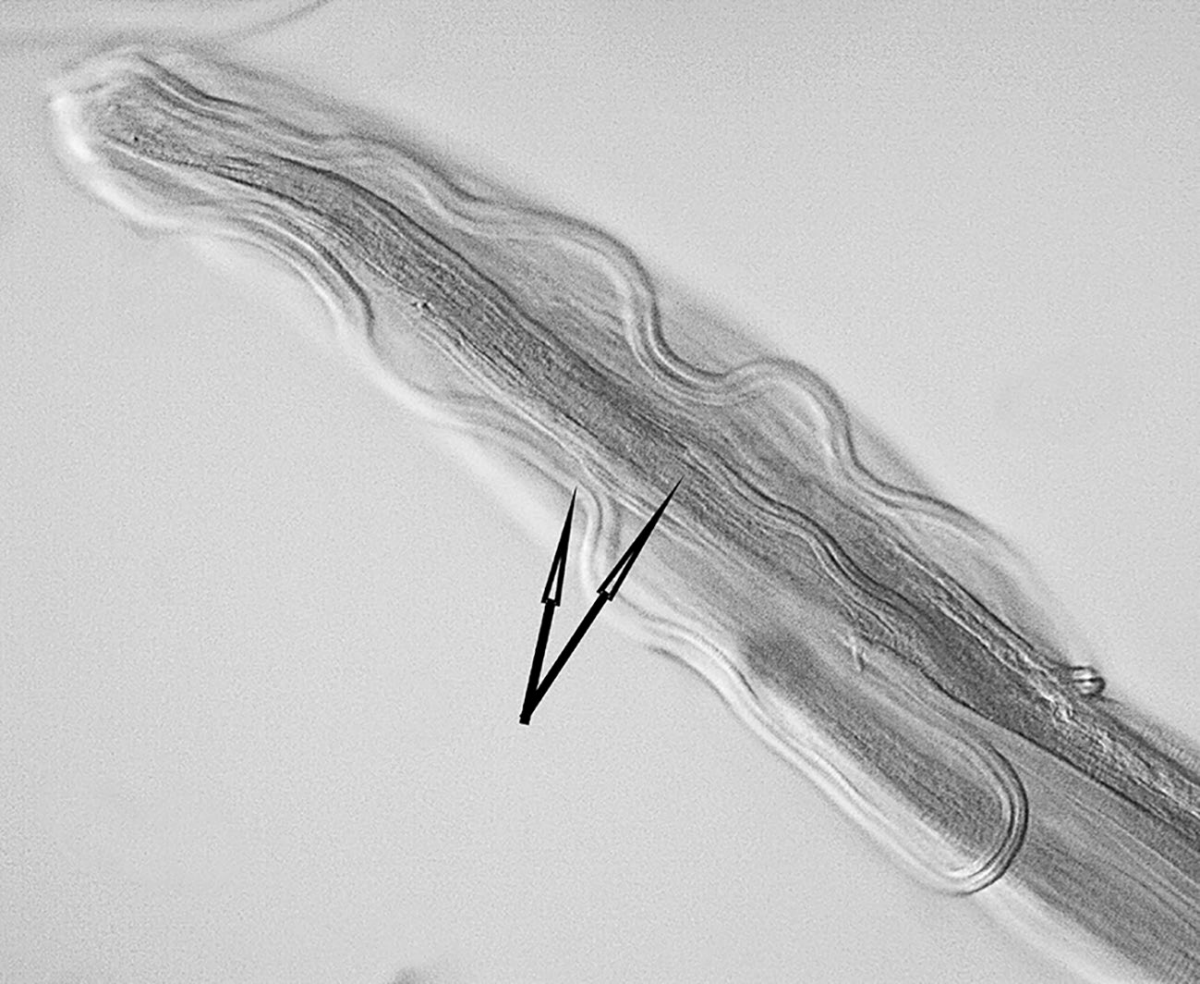
*Dispharynx* cf. *nasuta* from White Stork *Ciconia ciconia* (Poland), anterior end of coelom in female specimen. Scale: 100 μm. Arrows indicate the ends of the cuticular cordons.

**Fig. 3:**
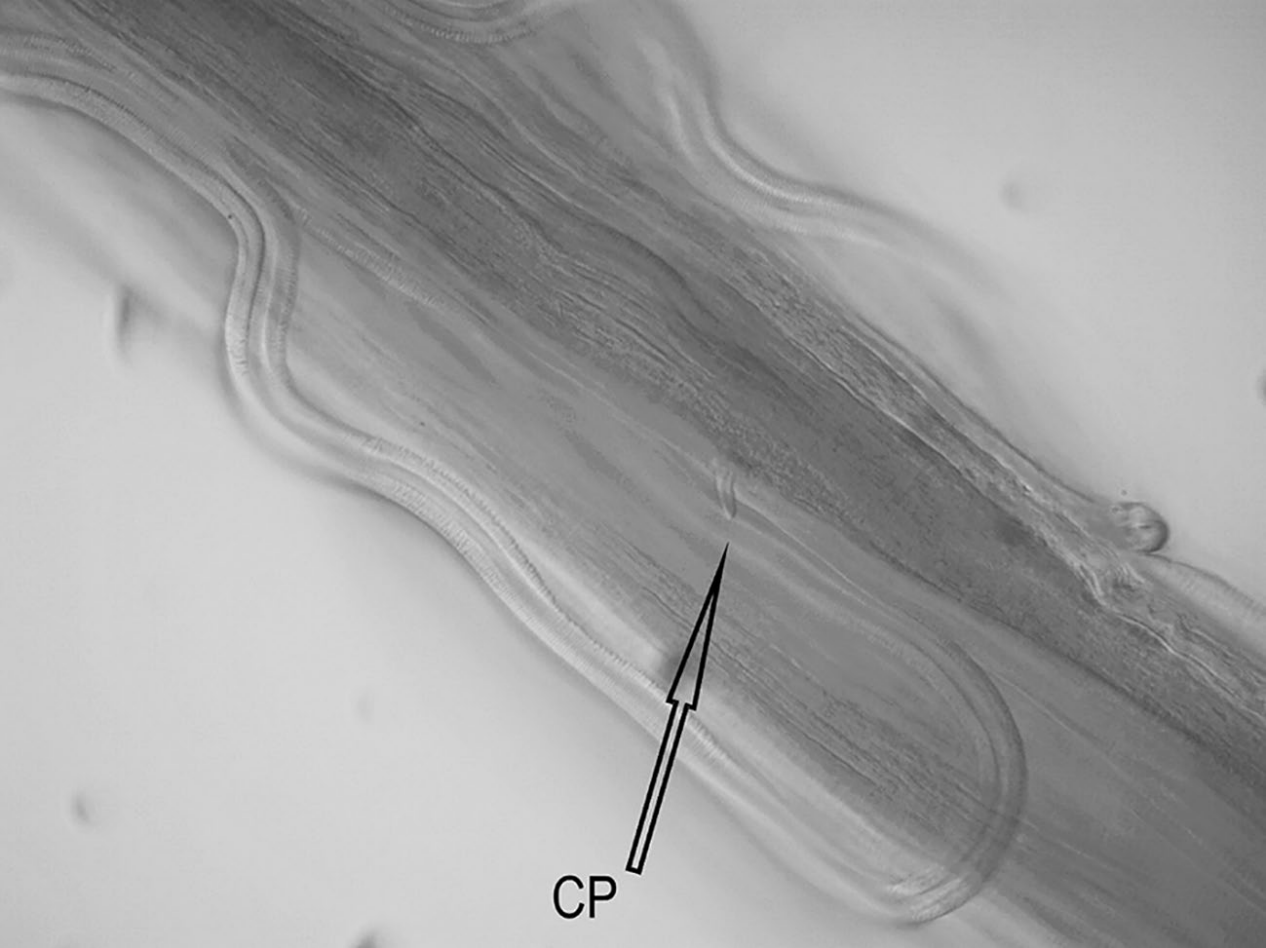
*Dispharynx* cf. *nasuta* from White Stork *Ciconia ciconia* (Poland), anterior end of coelom in female specimen. Scale: 100 μm. CP – cephalic papilla.

**Fig. 4:**
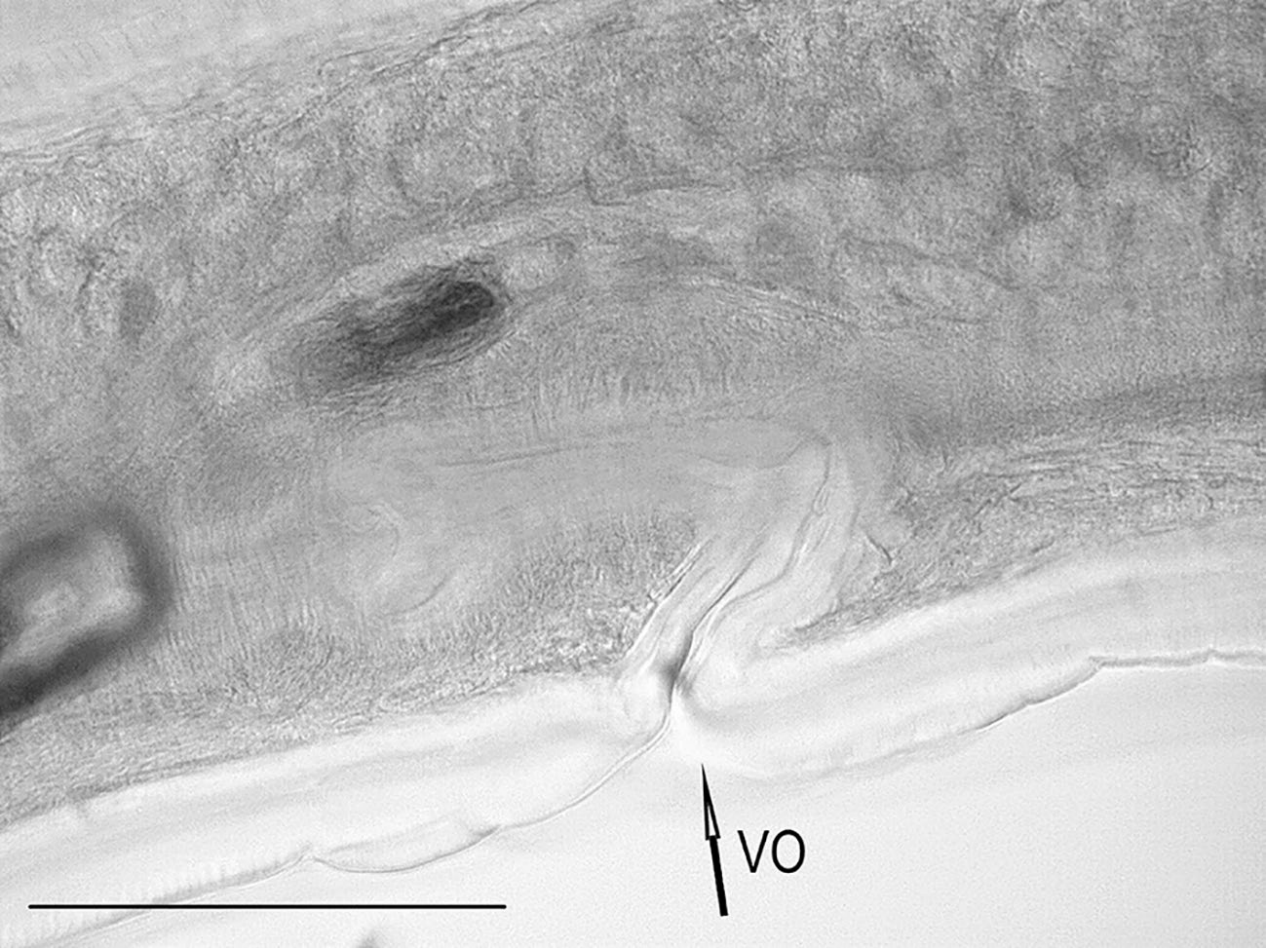
*Dispharynx* cf. *nasuta* from White Stork *Ciconia ciconia* (Poland), vicinity of vulva in female specimen. Scale: 100 μm. VO – vulvar opening.

**Fig. 5:**
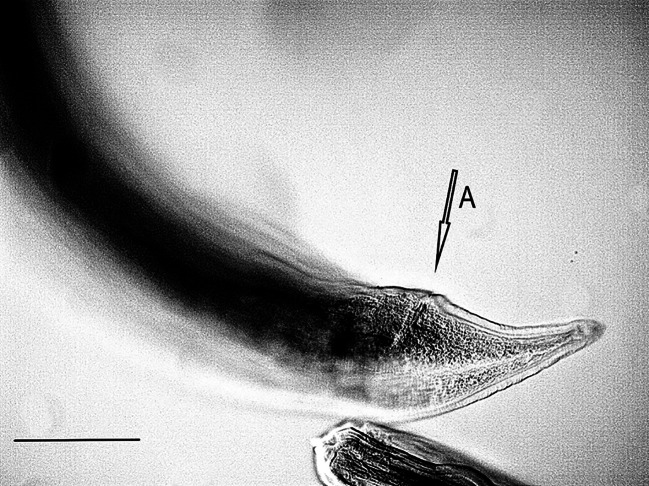
*Dispharynx* cf. *nasuta* from White Stork *Ciconia ciconia* (Poland), posterior end of coelom in female specimen. Scale: 100 μm. A – anus.

One female nematode assigned to the species *Dispharynx* cf. *nasuta* (Rudolphi, 1819) Stiles & Hassall, 1920 (Acuariidae) was found in the study material. The appearance of the body of the female nematode is consistent with descriptions available in keys and original works^4,20,21^^30^. Two pairs of S-shaped cuticular cordons run along the body, from the small labia at the buccal cavity. The descending branch of the cordon forms a loop, while the ascending part reaches one third of its length. There is a spike-shaped cephalic papilla between the ascending branches of the two cordons (Figs. 1, 2 and 3). No muscular or glandular oesophagus was observed in the female specimen. The vulval opening was located in the posterior part of the body. The appearance of the uterus and vulva is presented in the photograph in Fig. 4. The tail is pointed at the end (Fig. 5). No eggs were observed in the reproductive organs. The morphometric characteristics of the nematode are presented in Table 6.

**Table 6:** Morphometric characteristics of female *Dispharynx* cf. *nasuta* [mm].

Parameter	Our own material	Baruš et al^19^.	Smogorzhevskaya^20^	Pinto et al^21^.	Syrota et al^4^.	Al-Moussawi^31^	Hernandez-Urraca et al^30^.
Body length	5.25	3.47–9.21	3.4–3.6	5.134–6.902	5.4–7.4	3.998–5.876	3.96–7.2
Body width	0.23		0.24	0.306–0.578	0.376–0.650	0.252–0.415	0.322–0.646
Total cordon length	0.42	0.115–0.56		0.115–0.252	0.435–0.979	0.115–0.227	
Length of buccal capsule	0.1	0.09–0.14		0.097–0.119	0.089–0.129	0.094–0.127	
Distance from vulva to end of body	1.52	1.0–1.66.0.66	1.079	0.966–1.386	0.970–1.594	0.756–1.140	0.875–1.284
Tail length	0.135		0.128	0.08–0.126	0.119–0.217	0.118–0.140	

### Identification of cestode *Dictymetra riccii* (Fuhrmann & Baer, 1943) Clark, 1952

**Fig. 6:**
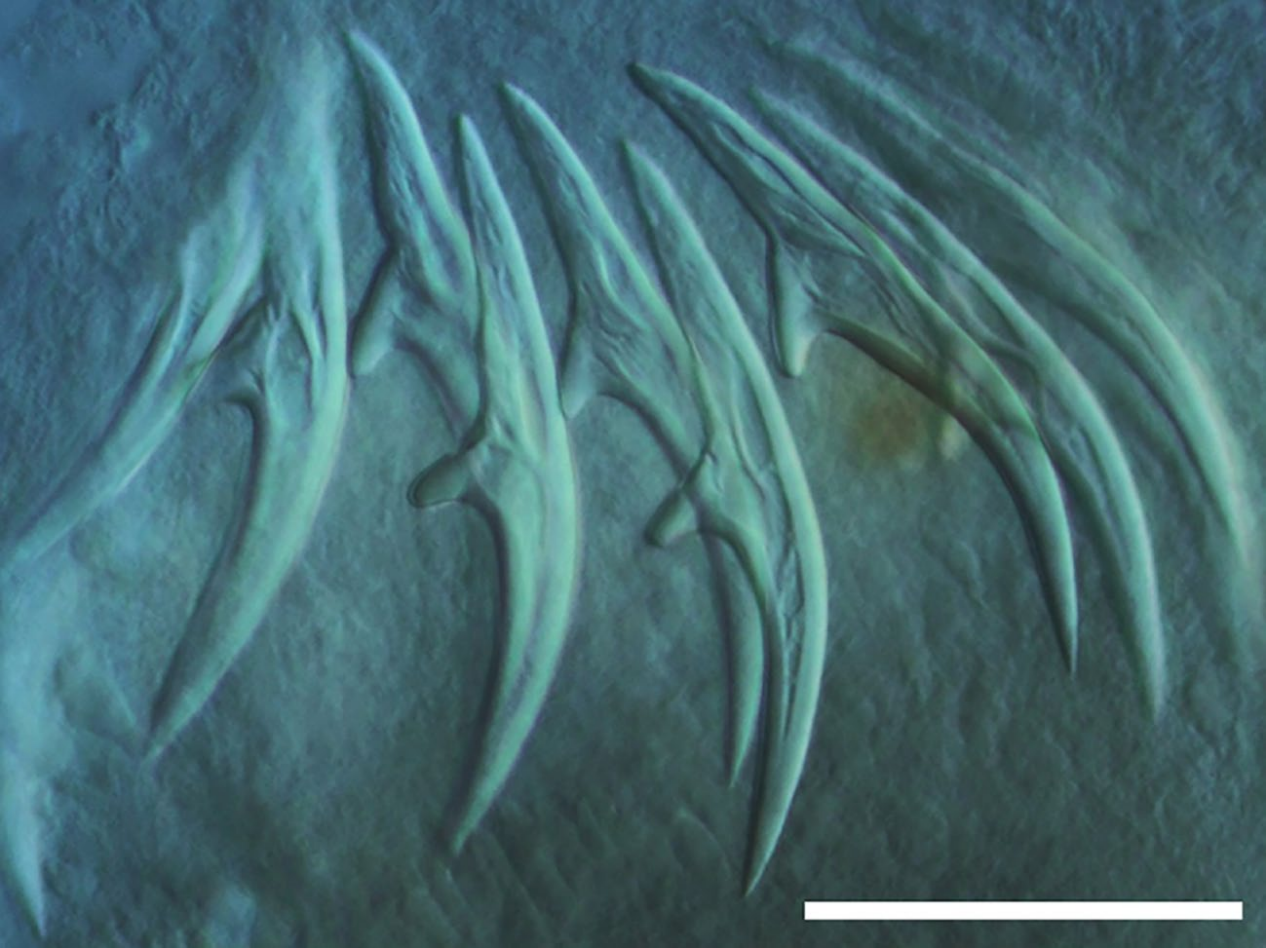
Rostelar hooks of *Dictymetra riccii*. Scale bar: 50 μm.

**Fig. 7:**
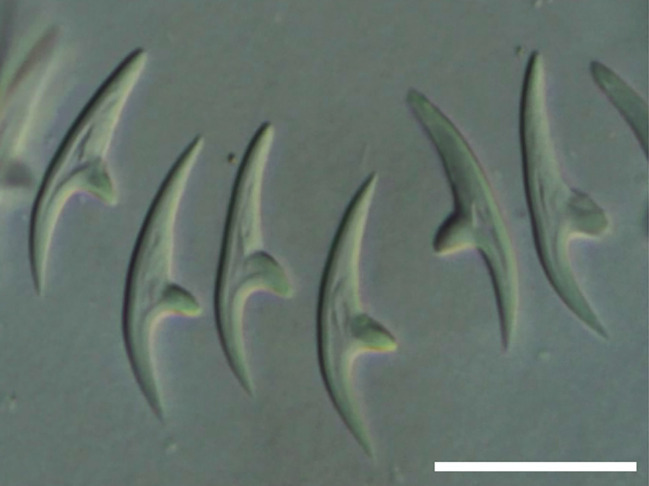
Rostelar hooks of *Dictymetra discoidea*. Scale bar: 25 μm.

Cestodes were identified by measuring the hooks and comparing them with literature data (Table 7)^32^. The shape of *Dictymetra riccii* hooks is shown in Fig. 6, and the shape of *Dictymetra discoidea* hooks is presented in Fig. 7.

**Table 7:** Characteristics of the Rostellar hooks of *Dictymetra riccii* (Fuhrmann & Bayer, 1943) Clark, 1952 [µm].

Author	Bona, 1975^32^					Present study				
Host species	*Ciconia abdimii* (= *Sphenorhynchus a*.)					*Ciconia ciconia*				
Geographic region	Congo					Poland				
Hooks	Anterior hooks		Posterior hooks		Δ	Anterior hooks		Posterior hooks		Δ
Length	mean	range	mean	range		mean	range	mean	range	
Total length	80.8	74.0‒86.5	84.5	77.0‒90.0	‒3.7	83.3 (*n* = 3)	79.0‒86.0	85.5 (*n* = 3)	84.0‒87.0	‒2.2
Blade	45.0	44.0‒46.0	39.2	36.0‒42.5	+ 5.8	47.4 (*n* = 3)	45.0‒49.0	41.9 (*n* = 3)	40.0‒44.0	+ 5.5
Handle	37.1	31.5‒41.0	46.0	42.5‒50.0	‒8.9	38.7 (*n* = 3)	37.0‒40.0	46.0 (*n* = 3)	44.0‒48.0	‒7.3

*Δ* Differences between mean values for anterior and posterior hooks, *n* Number of hooks measured.

### Results of molecular analysis of acanthocephalans

Two adult acanthocephalan specimens were detected in the storks. Based on their morphological characters, they were assigned to the genus *Centrorhynchus*.

In the molecular analysis of the acanthocephalans, high quality, identical 316 bp 18 S rRNA fragments were obtained for both specimens found in *C. ciconia*. Based on the sequence, we assigned the specimen to the species *Centrorhynchus magnus* (Fukui, 1929), with 97.77% similarity between the sequence obtained and the 18 S rRNA fragment of *C. magnus* deposited in GenBank under accession number LC713281. However, the same level of sequence similarity was found for the *Centrorhynchus* sp. record submitted under accession number AY830155.

## Discussion

The cestode *Dictymetra riccii*, the nematode *Dispharynx* cf. *nasuta*, and acanthocephalans are new to storks in Poland. There is also no information indicating that they have been found in White Storks in other parts of the world. The species richness recorded in our study is not great in comparison with previous parasitological studies of White Stork in Poland and around the world. For example, Sulgostowska and Czaplińska’s catalogue of trematodes^33^ names seven trematode species obtained in research conducted using from two to ten White Storks. Sitko and Heneberg^34^, based on analysis of the size of trematode populations in birds using farmland as feeding habitats in 1963–2020, provide interesting evidence of declines in trematode populations in White Stork. It is particularly worth noting the finding of a decline in the populations of trematode species which had previously been dominant in terms of prevalence and intensity of infection, i.e. *Tylodelphys excavata* and *Chaunocephalus ferox*^34^. The authors conclude that the causes of the losses observed are unclear, while at the same time pointing out the anti-helminthic activity of some agrochemicals.

In Poland, parasitological studies of storks have previously been carried out by researchers such as Gundłach^35^ and Michalczyk et al.^10^. The three trematode species and the cestode *Dictymetra discoidea* found in the present study had previously been recorded in Poland^36^. Previous research in Poland has also shown the presence of other trematodes in White Stork: *Bilharziella polonica* (Kowalewski, 1895) Looss, 1899 in the hepatic blood vessels, *Ignavia ciconiae* Sulgostowska, 1964 in the kidney (catalogue of trematodes), a single specimen of the trematode *Pegosomum spiniferum* Ratz, 1903 in the liver (study by Gundłach^35^ cited in Okulewicz^37^, and *Cyathostoma variegatum* (Creplin, 1849) in the trachea^38^. Our study in north-eastern Poland showed six helminth taxa that were not recorded in south-eastern Poland by Michalczyk et al.^10^, whereas the authors cited found three species of helminths in south-eastern Poland which were not present in our storks: the nematode *Syngamus trachea* (Montagu, 1811) and cestodes *Choanotaenia infundibulum* (Bloch, 1779) and *Raillietina tetragona* (Molin, 1858). *Chaunocephalus ferox* and *Cathaemasia hians* have been recorded in both north-eastern and south-eastern Poland.

Trematodes recorded outside of Poland include *Tylodelphys excavata* in Germany, Czechia, and Turkey^5,39,40^, *Tylodelphys clavata* (von Nordmann, 1832) in Turkey^5^, *Echinoparyphium* sp. and *Stephanoprora* (*Monilifer*) *spinulosa* (Rudolphi, 1809) in Germany and Turkey^5,40^, *Bilharziella polonica* in Czechia^40^, *Cathaemasia hians* in Germany, Czechia, and Slovakia^39–41^, *Chaunocephalus ferox* in numerous locations, such as Czechia, Turkey, Germany, Italy, Spain, Hungary, Ukraine, Austria, Belarus, Britain, Bulgaria, Moldova, Slovakia, Sweden, and Switzerland^5,8,13,39,40,42–44^, *Apharyngostrigea cornu* (Zeder, 1800) Ciurea, 1927, *Duboisia syriaca* (Dubois, 1934), *Echinochasmus spinulosus* (Rudolphi, 1808), *Echinostoma sudanense* Odhner, 1911, and *Stomylotrema pictum* (Creplin, 1837) in Czechia^40^, *Echinostoma revolutum* (Fröhlich, 1802) in Germany and Czechia^39,40^, and *Ignavia ciconiae* and *Brachylaimus* sp. in Germany^39^. Cestodes found in White Stork include *Dictymetra discoidea* (Beneden, 1868) and Hymenolepididae sp. in Germany^39^ and *Dictymetra discoidea* and *Schistocephalus solidus* (Müller, 1776) in Turkey^5^. Nematodes previously recorded in White Stork include *Contracaecum microcephalum* (Rudolphi, 1809), *Capillaria* sp., *Cyathostoma verrucosum* (Hovorka & Macko, 1959), *Excisa excisa* (Molin, 1860) Chabaud, 1958, *Syngamus palustris* Ryjikov, 1969, *S. trachea*, *Syncuaria ciconiae* Gilbert, 1927, *Paronchocerca ciconiarum* Peters, 1936, and *Tetrameres* sp^5,39,40,45^.

According to Dimitrova et al.^46^, the cestode species new to Poland found in our study, *Dictymetra riccii*, has previously been found in Africa (Ethiopia and Democratic Republic of the Congo) in *Ciconia abdimii* Lichtenstein, 1823.

The finding of *Dispharynx* cf. *nasuta* is the first record of this parasite species in White Stork *Ciconia ciconia*. Table 6 presents the dimensions of nematodes isolated from Common Pheasant, Himalayan Bulbul, Red-rumped Parrot, Common Crane, and Common Starling. The morphometric data of *Dispharynx* cf. *nasuta* to some extent correlate with the measurements reported by other authors. The most similar measurements were reported by Syrota et al.^4^, who described *D. nasuta* specimens isolated from Common Crane (*Grus grus* Linnaeus, 1758). However, since our discovery is the first record of a nematode of the genus *Dispharynx* in White Stork, the literature provides no available measurements for comparison. The life cycle of *D. nasuta* is complex and involves intermediate hosts, which are various species of terrestrial isopods (e.g. Common Rough Woodlouse *Porcellio scaber* Latreille, 1804, Common Pill-bug *Armadillidium vulgare* Latreille, 1804, the genus *Oniscus*, *Venezillo evergladensis* Schultz, 1963, and *Oscelloscia floridana* (Van Name, 1940)). The definitive hosts are birds of the orders Galliformes (landfowl), Gruiformes, Charadriiformes (shorebirds), Columbiformes (pigeons and doves), Passeriformes (passerines), Psittaciformes (parrots), Cuculiformes (cuckoos), Coraciiformes, and Piciformes^30,31,47^. In Poland, the occurrence of *D. nasuta* has been described in the rook (*Corvus frugilegus* Linnaeus, 1758), European Robin (*Erithacus rubecula* Linnaeus, 1758), Eurasian Jay (*Garrulus glandarius* (Linnaeus, 1758)), and Common Starling (*Sturnus vulgaris* Linnaeus, 1758)^36^. *D. nasuta* is regarded as the most pathogenic species of its genus, and infection with it leads to dispharynxiasis, manifested as anorexia, weight loss, and weakness. The nematodes settle in the glands of the proventriculus, where they induce extensive inflammation of the mucosa. This leads to thickening of the mucosa and gastrointestinal obstruction. Other pathomorphological changes in the proventriculus wall include ulcers and gland destruction. Dispharynxiasis can end in the death of the birds^30,47^.

Acanthocephalans were recorded in White Stork for the first time in our study. We identified them to the genus *Centrorhynchus* of the family Centrorhynchidae. The spectrum of acanthocephalan species for which the White Stork can function as a host is as yet unknown. Acanthocephalans of the family Centrorhynchidae are cosmopolitan and commonly found in birds and mammals. Acanthocephalans of the genus *Centrorhynchus* are known in Poland: *Centrorhynchus aluconis* (Muller, 1780) has been found in raptors such as Eurasian Goshawk *Accipiter gentilis* Linnaeus, 1758 and Common Buzzard *Buteo buteo* (Linnaeus, 1758) and in owls *Strix aluco* Linnaeus 1758 and *S. uralensis* Pallas, 1771, while *Centrorhynchus buteonis* (Schrank, 1788) has been recorded in Poland in *Buteo buteo* (Linnaeus, 1758)^36^. In reptiles, *C. alucois* has been found in larval (cystacanth) form in Slow Worm *Anguis fragilis* Linnaeus, 1758, Sand Lizard *Lacerta agilis* Linnaeus, 1758, and Common European Viper *Vipera berus* (Linnaeus, 1758)^36^. In Slovakia, Komorová et al.^47^ reported an apparent difference in the acanthocephalan species spectrum between birds of prey and owls. *C. aluconis* was the most widespread species among birds, while *C. buteonis* and *Centrorhynchus globocaudatus* (Zeder, 1800) were found less often, in different species^48^.

The presence of *Centrorhynchus magnus* Fukui, 1929 in *C. ciconia* has not yet been reported in the genetic databases. According to Lisitsyna and Greben^49^, acanthocephalans of the genus *Centrorhynchus* are mainly found in birds of prey and owls, and rarely in birds of other groups. The record submitted to GenBank under accession number LC713281 indicates that *C. magnus* was found in a Black-crowned Night Heron (*Nycticorax nycticorax*) in Japan^50^. According to the host–parasite database hosted by the Natural History Museum in the UK (https://www.nhm.ac.uk), *C. magnus* specimens have also been identified in three other bird species studied in Japan: Eurasian Sparrowhawk (*Accipiter nisus nisosimilis* (Tickell, 1833)), Japanese Buzzard (*Buteo buteo japonicus*), and Black-eared Kite (*Milvus migrans lineatus* (J.E. Gray, 1831))^51,52^. However, *C. magnus* specimens were also identified in birds in the Danube delta^53^ and in Odesa Region (Vylkove)^53,54^. The sequences identified in the present study were identical to those of an unidentified species of the genus *Centrorhynchus* (GenBank, acc. no. AY830155) identified in Peregrine Falcon (*Falco peregrinus* Tunstall, 1771), another bird of prey. Therefore, the finding of *C. magnus* in *C. ciconia* requires further investigation, possibly using high-throughput sequencing technologies, as acanthocephalans parasitize a diverse range of definitive bird hosts and various crustaceans as intermediate hosts. Thus, the consumption of infected invertebrates and lower vertebrates by *C. ciconia* may complete the life cycle of *C. magnus.*

The trematodes *Chaunocephalus ferox* and *Cathaemasia hians* recorded in the study are described by Michalczyk et al.^10^ as not endemic to the study area (south-eastern Poland). Trematodes *C. ferox* and *C. hians* have been recorded in Poland in White Stork and Black Stork (*C. nigra*). *C. ferox* larvae have not been found in Poland^36^, but the larvae of *C. hians* have previously been recorded in Poland and other countries of Central and Eastern Europe in snails *Anisus calculiformis* (Sandberger, 1874), *Bathyomphalus contortus* Linnaeus, 1758, *Lymnaea stagnalis* (Linnaeus, 1758), *Planorbis planorbis* (Linnaeus, 1758), and *Stagnicola palustris* (O.F. Müller, 1774) (cercariae)^36,55^, as well as in *Bombina bombina* (Linnaeus, 1761), *Rana esculenta* (Linnaeus, 1758), *R. lessonae* (Camerano, 1882), and *R. ridibunda* (Pallas, 1771) (metacercariae)^36^. The trematode *Chaunocephalus ferox* is known for its pathogenicity^8,10,13,56^. It is believed to be a typical parasite of White Stork and is usually the dominant species in the helminth community, with high intensity and frequency^8,40^. Apart from White Stork, it has also been found in Black Stork (*Ciconia nigra* (Linnaeus, 1758)), Eurasian Bittern (*Botaurus stellaris* (Linnaeus, 1758)), Black-necked Stork (*Xenorhynchus asiatius* (Latham, 1790)), and Asian Openbill (*Anastomus oscitans* (Boddaert, 1783))^13^. Clinical symptoms of infection with *C. ferox* include weakness, diarrhoea, severe intestinal catarrh and enteritis, and cachexia^13^. Trematodes of this species induce the formation of closed granulomas in the intestinal lumen, each containing two^8,13^. The histopathological image at the sites of penetration of the trematodes shows tissue destruction with haemorrhaging and thickening of the mucosal, submucosal, and muscular layers. A very common phenomenon is visible lymphocytes, histiocytes, and heterophils accumulated around the parasites. Older intestinal damage involves tissue necrosis^8^. Pathological lesions in the intestinal wall most likely inhibit nutrient absorption from food. Therefore, infection with this parasite affects flight efficiency and the capacity for a predatory way of life. The degree of pathogenicity of *C. ferox* depends on the number of parasites present in the host and on whether the infection is accompanied by other stress factors^8^. Sitko and Heneberg^40^ noted weight loss and higher mortality due to ulcers and intestinal catarrh in storks in which *Tylodelphys excavata* co-occurred with *C. ferox*, in comparison to those infected with *C. ferox* alone. However, infection with these helminth species does not always cause such devastating illness, as reported by Girisgin et al.^5^. In a study by Michalczyk et al.^10^, *C. ferox* was the most common among all parasites, i.e. in 11 storks.

The prevalence of helminths in our study was 32.8%, with cestodes noted most often, and among these, *Dictymetra discoidea* was most common (prev. 19.7%). Prevalence of nematodes was lowest among all helminths. Michalczyk et al.^10^ examined storks at nesting sites in Poland. The storks were aged three weeks to five years, but the vast majority (about two thirds) were between three and 15 weeks old. The prevalence of helminths in that study was close to 50%, which was somewhat higher than in the present study. The most commonly detected parasite in that study was the trematode *Chaunocephalus ferox.* The difference between the occurrence of cestodes and trematodes in our study and the study by Michalczyk et al.^10^ is most likely linked to the age of the birds examined (see the discussion below). Michalczyk et al.^10^ found nematodes in only two storks; these were of the species *Syngamus trachea*. Sitko & Heneberg^40^, on the basis of a long-term study of 91 White Storks (1962–2013), confirmed that *C. ferox*, *Tylodelphys excavata*, and *D. discoidea* are usually the dominant species in various European countries. In Czechia, the prevalence of these helminths was > 34% for both *C. ferox* and *T. excavata* and 25% for *D. discoidea*. In Turkey, eight helminth species were found in 18 storks (seven males and 11 females) examined in the years 2009–2015, with a prevalence of 94.44%^5^. The dominant species in that study in terms of prevalence were the cestode *Dictymetra discoidea* (> 38%) and the trematode *C. ferox* (> 37%), similar to the results of the present study, although in our study the prevalence of these parasites was lower (Table 3). Girisgin et al.^5^ found no pathological changes caused by helminths in the internal organs of the storks. Girisgin et al.^5^ recorded the presence of only one nematode specimen, of the species *Syncuaria ciconiae*. The intensity of infection of helminths in the present study was 12.2 (1–60), with dominance of *T. excavata*. Sitko & Heneberg^40^ reported that trematodes *T. excavata* were present with the greatest intensity in White Stork, followed by *C. ferox* and *D. discoidea*. In the study by Girisgin et al.^5^, high intensity of infection was observed for cestodes *D. discoidea* and *Schistocephalus solidus* and – as in the present study – for the trematode *Tylodelphys excavata*.

Comparison of ecological indices (Simpson’s, Berger–Parker, and Brillouin) to the structure of communities in other bird species in which such analyses have been performed (e.g. Tufted Duck, Greater Scaup, and marine ducks^57,58^ shows that the component community is moderately diverse, while the biodiversity of the infracommunities is low. The Berger–Parker dominance index indicates that the component community is not strongly dominated by a single species (*D. discoidea*) and remains at a level of about 50% (Berger–Parker index = 0.485). Sitko & Heneberg^40^ reported that the Berger–Parker dominance index was only 0.24 when calculated on the basis of prevalence, but 0.67 when based on frequency (*C. ciconia*). The dominant species in that study were trematodes *C. ferox* and *T excavata*.

We observed an increase in the species richness of helminths from April to July, accompanied by a decrease in the intensity of infection. However, this may be associated with the sample sizes in these months (more storks were examined in July than in other months), and definitive conclusions cannot be drawn from this analysis. Storks arrive in Poland in March, returning from wintering grounds located in southern Africa. For this reason, the beginning of the study, i.e. as early as possible after the storks’ arrival at their breeding grounds, is an important moment. At the start of our study, in April, we detected *C. ferox*, *C. hians* and *D. discoidea*. The trematode *C. ferox* was also present in subsequent months of the study. Trematodes *C. hians*, however, were not found in subsequent months in storks, which may indicate that this trematode was introduced during migration from the birds’ wintering grounds. *C. hians* is a specific parasite of storks and is spread throughout the Palaearctic realm and Central Africa. According to data collected by Sitko et al.^59^, the first intermediate hosts in the life cycle of this trematode are *Planorbis planorbis* and Lymnaeidae, and the second intermediate hosts are frogs (*Bombina*, *Bufo*, and *Rana*). The life cycle of *C. hians* is completed in warm climates^2^.

The cestode *D. discoidea* was noted in April and was also present in the storks in later months. The presence of *D. discoidea* (also described using the synonym *Anomotaenia discoidea* (Beneden, 1868, Fuhrmann, 1908)) has been recorded in Europe and Africa. In Poland, it has previously been recorded in White Stork and Black Stork, and the highest intensity of infection was 32 cestodes per host. Dimtrova et al.^46^ conducted a review of cestodes of birds recorded in Africa, referring to data on the occurrence of *D. discoidea* in Sudan, Egypt, and Morocco. In addition to *D. discoidea*, cestodes *Oschmarinolepis microcephala* (Rudolphi, 1819) and *Paraoschmarinolepis multiformis* (Creplin, 1829) have also been found in White Stork in Africa^46^.

In our study, the highest prevalence was noted in the group of female chicks (66.7%) and adult females (44.4%) (Table 5). Intensity of infection was highest in one female chick (60 trematodes *T. excavata*). Michalczyk et al.^10^ found that the prevalence of helminths was highest in the group of birds aged 3–4 weeks (84.6%), in comparison with older age groups; the vast majority of birds in the youngest age group were infected with *C. ferox*. Prevalence of infection of adult birds above the age of two years in the study by Michalczyk et al.^10^ was less than 40% (38.46%), and thus similar to the level noted in the present study in adult storks. In the study by Michalczyk et al.^10^, cestodes were not present at all in storks up to the age of four weeks and were found only sporadically in older storks. We observed a similar tendency in our study, i.e. statistically significantly higher prevalence of cestodes in adult storks than in juveniles, while in chicks there were no cestodes at all. Sitko & Heneberg^40^ reported that juvenile White Storks were affected by a higher parasite load than adults, with high intensity of infection with *Chaunocephalus ferox* and *Tylodelphys excavata* occasionally detected in juvenile hosts. There were no significant differences between the infection of female and male storks by helminths in our study. Sitko & Heneberg^40^ reported similar relative prevalence and frequency of helminths in male and female *C. ciconia*.

## Conclusion

The community structure of helminths of White Stork in north-eastern Poland was described in this study. The helminth fauna of White Stork consists of seven taxa, including three that are newly described in this paper in White Stork: *Dictymetra riccii*, *Dispharynx* cf. *nasuta* and *Centrorhynchus* sp. Among infected storks, which constituted more than 30% of those examined, the dominant parasite species was the cestode *Dictymetra discoidea*, whose prevalence was significantly higher in adult storks than in juveniles. Nearly 10% prevalence was noted for the trematode *Chaunocephalus ferox*, which is known for its pathogenicity. The results may be significant for evaluation of the condition of the White Stork population.

## Data Availability

The datasets generated and analysed during the study are available in the NCBI database: (https:/www.ncbi.nlm.nih.gov/nuccore/PX446822) (accession number PX446822.1).
